# Draft Genome Sequence of a Probiotic Strain, *Lactobacillus fermentum* UCO-979C

**DOI:** 10.1128/genomeA.01439-15

**Published:** 2015-12-10

**Authors:** Andrey V. Karlyshev, Julio Villena, Carlos Gonzalez, Leonardo Albarracin, Javier Barros, Apolinaria Garcia

**Affiliations:** a Biomolecular Sciences Department, School of Life Sciences, Pharmacy and Chemistry, Faculty of Science, Engineering and Computing, Kingston University, Kingston upon Thames, United Kingdom; b Reference Centre for Lactobacilli (CERELA-CONICET), Laboratory of Immunobiotechnology, San Miguel de Tucumán, Tucumán, Argentina; c Microbiology Department, Faculty of Biological Sciences, University of Concepcion, Concepción, Chile

## Abstract

This report describes a draft genome sequence of *Lactobacillus fermentum* strain UCO-979C. The reads generated by a Ion Torrent PGM were assembled into contigs, with a total size of 2.01 Mb. The data were annotated using the NCBI GenBank and RAST servers. Specific features of the genome are highlighted.

## GENOME ANNOUNCEMENT

A gastric probiotic, *Lactobacillus fermentum* strain UCO-979C, isolated in 2007 (1), revealed anti-*Helicobacter pylori* activity inhibiting pathogen adhesion and modulating host inflammatory response (A. García, F. Márquez, S. Pineda, K. Navarro, E. Pastene, M. Quezada, K. Henríquez, A.V. Karlyshev, J. Villena, C. González, unpublished data). A draft genome sequence reported here was generated using an Ion Torrent PGM. The sequencing reads generated on Chip 314 version 2 were assembled by two Torrent Assembler plugins (MIRA and SPAdes) and the CLC Genomics Workbench (version 7.5) *de novo* assembler, and then merged using CISA contig integrator (2) to produce 108 contigs (1 kb to 137.2 kb), with a total size of 2,011,828 nucleotides (nt) (19.46× coverage). Both the genome size and G+C content (51.9%) are in a good agreement with published data for *L. fermentum* (2.1 to 2.33 Mb and 50.56 to 51.50%, respectively). Strain UCO-979C showed 100% identity (100% coverage) with *L. fermentum* strain IFO 3956 16S rRNA gene and almost 100% identity with this gene in other *L. fermentum* strains. A similar result was obtained when using read mapping onto DNA gyrase-encoding genes (*gyrA* and *gyrB*).

The genome sequence was annotated using the NCBI GenBank annotation pipeline and RAST genome annotation server (3). A gene encoding a collagen-binding protein (CBP) showing the highest level of amino sequence similarity with a CBP from *L. casei* (sequence ID WP_012552829.1) was found. The CBP of strain UCO-979C is also homologous to CBP from *L. fermentum* 3872 (4, 5) (accession numbers CP011537.1 and CP011536.1). Interestingly, the *cbp* gene is found on a contig containing a plasmid replication control protein and a toxin-antitoxin pair required for stable plasmid maintenance, as we also found in a previously described plasmid, pLF3872, of strain 3872 (5). The only other sequenced strain of *L. fermentum* with a *cbp*-like gene is NB-22 (6), but the derived protein contains just one copy of the repeating unit. A gene encoding a fibronectin-binding protein A identical to that of *L. fermentum* NB-22 (GenBank accession no. ESS01578.1) (6) was also detected. A number of other genes identical or highly similar to those in NB-22 were also found.

Three contigs with a total size of 40 kb reveal significant similarities with the plasmids found in other *Lactobacillus* spp., suggesting the presence of at least one plasmid in strain UCO-979C. In particular, a 16.7-kb contig showed 96% identity (51% coverage) to a *Lactobacillus salivarius* UCC118 plasmid, pSF118-20 (accession no. AF488831.1), a 5.5-kb contig showed 99% identity (71% coverage) to plasmid 2 of *Lactobacillus paracasei* subsp. *paracasei* 8700:2 (accession no. CP002393.1), and a 17.2-kb contig showed 99% identity (99% query coverage) to *L. casei* strain Zhang plasmid plca36 (accession no. CP000935.1).

The closest genome sequences are those of strains NB-22 (among draft genome sequences) and 3872 (among completely sequenced genomes). Genes present in 3872 but missing in UCO-979C included amino acid transporter proteins, various membrane and hypothetical proteins, daunorubicin resistance protein, arsenical pump membrane protein, manganese transporter, various adhesins a gene cluster encoding allantoin permease, crystallin; and ribokinase; two prophages, and a number of transposases.

### Nucleotide sequence accession numbers.

This whole-genome shotgun project has been deposited at DDBJ/EMBL/GenBank under the accession no. LJWZ00000000. The version described in this paper is version LJWZ01000000.
